# Quantitative Trait Loci Mapping for Yield and Related Traits in Cowpea

**DOI:** 10.3390/genes16030247

**Published:** 2025-02-21

**Authors:** Abdoul Moumouni Iro Sodo, Patrick Obia Ongom, Christian Fatokun, Bunmi Olasanmi, Ibnou Dieng, Ousmane Boukar

**Affiliations:** 1 International Institute of Tropical Agriculture (IITA), PMB 5320, Oyo Road, Ibadan 200001, Nigeria; a.iro-sodo@cgiar.org (A.M.I.S.); c.fatokun@cgiar.org (C.F.); i.dieng@cgiar.org (I.D.); 2 Department of Crop and Horticultural Sciences, Pan African University Life and Earth Sciences Institute (Including Health and Agriculture), University of Ibadan, Ibadan 200284, Nigeria; 3 International Institute of Tropical Agriculture (IITA), PMB 3112, Sabo Bakin Zuwo Road, Kano 700223, Nigeria; o.boukar@cgiar.org; 4 Department of Crop and Horticultural Sciences, University of Ibadan, Ibadan 200284, Nigeria; bunminadeco@yahoo.com

**Keywords:** *Vigna unguiculata*, high-yielding varieties, grain yield traits, DArT technology, QTL mapping, RIL population

## Abstract

**Background/Objectives:** Cowpea is a major source of dietary protein and plays a key role in sustainable agriculture across sub-Saharan Africa (SSA), Asia, and Latin America. Research efforts have focused mainly on enhancing productivity through higher yield and resistance to biotic and abiotic stresses in cowpea. Understanding the genetic basis of yield and associated agronomic traits is crucial for improving crop productivity. This study aims to identify quantitative trait loci (QTL) associated with grain yield and related traits in cowpea under regular rainfed conditions. **Methods:** We developed a set of 316 F6:7 recombinant inbred lines (RILs) mapping populations derived from a cross between RP270 and CB27 using a single-seed descent breeding method. The RILs and their two parental lines were evaluated in the field for two years, 2022 and 2023, at the International Institute of Tropical Agriculture (IITA) in Ibadan, Nigeria. The cowpea mid-density genotyping panel consisting of 2602 quality DArTag single nucleotide polymorphisms (SNPs) was used to genotype the RIL population. **Results:** Seven major QTLs, each explaining ≥10% of phenotypic variance, were detected for 100-seed weight, number of days to flower, number of pods per plant, number of branches per plant, and number of peduncles per plant. Putative genes associated with yield and related traits were identified within significant flanking markers. Further efforts to validate these loci will help to better understand their roles in yield and associated traits in cowpea.

## 1. Introduction

Cowpea (*Vigna unguiculata* [L.] Walp.) is a diploid species (2n = 22) with an estimated nuclear genome size of 640.6 Mbp [1]. It is an important grain legume in SSA, where it is widely cultivated and consumed [2]. It is especially well-suited to the SSA’s dry savannah and Sahel regions, where other crops often perform poorly due to water stress caused by unpredictable and short-duration rainfall and low fertility soils [2,3]. Cowpea is important for diverse reasons. This crop has good nutritional values and is a valuable cash crop in semi-arid locations [4]. It plays a crucial role in human nutrition due to the high dietary value of its grain, which contains 23–32% quality protein (rich in lysine, tryptophan), and substantial amounts of minerals and vitamins compared to other crops [5]. Low crop yields in SSA are due largely to poor soil fertility, high temperature, drought caused by irregular rainfall coupled with lack of irrigation, cultivation of unimproved varieties, inadequate cultural practices, diseases, parasitic weeds, and pests [6]. Developing better genotypes by selecting high-yielding varieties is a crucial long-term strategy to combat low crop yields in SSA. To enhance food security and address nutrient deficiencies in the dry savannah regions of SSA, it is important to increase cowpea production by developing improved resilient varieties with consumer-preferred traits. To develop improved high-yielding cowpea varieties, an understanding of the genetic basis of yield and associated traits is essential [7].

Molecular genetic tools, where available, can contribute positively to the development of improved cowpea varieties. For example, identifying single nucleotide polymorphism (SNP) markers associated with desirable traits would facilitate the rapid development of better-performing genotypes through marker-assisted selection (MAS) in cowpea breeding programs. Studies have been conducted using QTL mapping to understand the genetic mechanisms associated with yield and related traits, as well as resistance to some abiotic and biotic stresses in cowpea. For instance, QTLs associated with days to flowering [8,9,10,11], pod length [12,13], seed number per pod [14], number of peduncles per plant, number of pods per plant, 100-seed weight [13], peduncle length [9], days to maturity [15], resistance to *Aphis craccivora* [16,17], and resistance to root-knot nematodes [18,19] and Striga gesnerioides [20] have been reported. Despite the identification of several QTLs, only a few have been found useful due to their general small effects and significant environmental influences associated with complex traits. Over the past decade, the cost of genome sequencing has decreased significantly due to technological advancements. This reduction in genotyping costs has yielded the development of low- to high-density genotyping platforms for some crops. Due to cost concerns regarding these platforms, the focus has recently shifted to using still cheaper genotyping technologies such as the Diversity Arrays Technology (DArT) SNP marker systems due to their lower cost per data point, high throughput, and robust data generation within a short period. Garcia–Oliveira et al. [13] used the DArTseq markers platform for QTL mapping for grain yield-related cowpea traits. There has been good progress using DArT in diverse options tailored to address different breeding demands. One of the recently introduced sets of DArT options includes the targeted genotyping (DArTag) method, which enables genotyping using selected marker sets. The DArTag represents an alternative among the various targeted genotyping solutions developed by the DArT company. Utilizing DArTag makes it feasible to target any SNP (or a small indel) if some genomic sequence is available around the variant base. The DArTag provides cost-effectiveness and diminishes the burden of bioinformatics, making it well-suited for high-throughput scenarios [21]. This study aims to construct a medium-density genetic map for the identification of QTLs for yield and related traits using an RIL population. Using the annotated reference genome [22], we also identified putative genes within QTL regions that control yield and some yield-related traits. The QTLs and candidate genes uncovered in the current study hold the potential for enhancing genetic studies and breeding efforts in cowpea.

## 2. Materials and Methods

### 2.1. Plant Materials

A recombinant inbred line (RIL) population derived from a cross between RP270 (Region des Plateaux 270) and CB27 (California Blackeye 27), comprising 346 lines, was used in the present study. The line RP270 is a landrace identified for its seed coat traits and unique colorless eye following a genetic diversity study of germplasm lines collected from Togo [23], while CB27 (California black eye 27) is an improved variety from the USA. The two parental lines differ in seed coat texture, seed morphology, seed size, 100-seed weight, seed eye color, and number of days to flower. The seeds of line RP270 are white colored with a smooth coat texture, no eye color, and a relatively smaller size (approx. 100-seed weight: 15.5 g), while CB 27 seeds are also white, with black eyes, rough seed coat texture, and medium size (approx. 100-seed weight: 18.7 g). The F1 seeds were sown in pots placed in a glasshouse to generate seeds for F2. Each F2 plant was then advanced by single seed descent to F6:7 to constitute the set of 316 RILs used for this study.

### 2.2. Experimental Design and Phenotyping

The parental lines and RILs were evaluated using an incomplete lattice design with three replications at the research field of IITA (N 7.50250° E3.89411°), Ibadan, Nigeria, for two years. The seeds were sown on 13 October 2022 and 4 September 2023. Seeds were planted in plowed and harrowed plots, with each line in three rows of 2.0 m length, spacing of 1.0 m between rows, and 0.25 m within rows. The seeds were pretreated with hexachlorobenzene (Granox N-M Fungicide Seed Treatment Patch) at a 10 g/kg rate before sowing. Two seeds were sown per hole, and seedlings were thinned to one plant per stand two weeks after sowing. A compound fertilizer (NPK 15:15:15) was applied at a rate of 6 g per stand two weeks after planting. The plots were kept insect-free by spraying chlorpyriphos (Thermex 48EC) and lambda-cyhalothrin (Karate 5EC) with 2 l/ha and 1.5 l/ha, respectively, at the vegetative, flowering, pod formation, and pod filling stages. Manual weeding was carried out as necessary to ensure no adverse weed interference. Ten plants were randomly selected from each plot, and data for the following traits were recorded from them: number of days to first flower opening (NDFW), number of branches per plant (NBrch), number of peduncles per plant (Nped), peduncle length (PedLt), pod length (PodLt), number of pods per plant (NPod), number of seeds per pod (NSP), 100-seed weight (100SW), and grain yield (GY). Peduncle and pod lengths were recorded using a flexible measuring tape. Peduncle length was recorded by measuring the distance from the point of peduncle attachment to the stem node to where the first flower bud appeared. The number of seeds per pod was recorded on average from ten randomly selected mature, dry pods. After harvesting, the pods were dried in the glasshouse before being threshed. After threshing, the total seed weight and 100-seed weight were determined.

### 2.3. Statistical Analysis

Descriptive statistics, such as mean, range (maximum and minimum values), skewness, and kurtosis for all the traits, were estimated using R software version 4.2.3 [24]. The frequency distributions of all the measured traits were determined. Analysis of variance (ANOVA) was carried out on data for each year and both years combined using the best linear unbiased estimations (BLUE) generated following Lme4 [24] as follows:

Yijk = µ + Gg + yi + (Gg: yi) + Rij + R(blk) + εijk

where Yijk is the phenotypic value, µ is the grand mean, Gg is the effect of the g genotypes, yi is the effect of the ith planting year; Gg: yi is the genotype × year interaction of genotype g and year i, Rij is the effect of the jth replicate in the year ith, R (blk) is the effect of the kth incomplete block within the jth replicate, and εijk is the experimental error. Phenotypic variation measured for each of the traits was evaluated using the formulae described previously by Burton [25]. The PCV and GCV were calculated and classified into three classes: <10% (Low), 10–20% (Moderate), and >20% (High).

$$\mathrm{Phenotypic}\;\mathrm{Coefficient}\;\mathrm{of}\;\mathrm{Variance}\left(\mathrm{PCV}\right)=\frac{\sqrt{Phenotype\;variance}}{\mathrm{Mean}}\;\times \;100$$

$$\mathrm{Genotypic}\;\mathrm{Coefficient}\;\mathrm{of}\;\mathrm{Variance}\left(\mathrm{GCV}\right)=\frac{\sqrt{Genotype\;variance}}{\mathrm{Mean}}\;\times \;100$$

$$\mathrm{Environmental}\;\mathrm{coefficient}\;\mathrm{of}\;\mathrm{variation}(\mathrm{ECV})=\frac{\sqrt{Environment\;variance}}{\mathrm{Mean}}\;\times \;100$$

Heritability in a broad sense was estimated as the ratio of genetic variance to the phenotypic variance and categorized according to Johnson et al. [26] into three classes: <30% (Low), 31–60% (Medium), and >60% (High).

(1) $$Heritability\left(broad\;sense\right)=\frac{Genotype\;variance}{Phenotype\;variance}\times 100$$

Genetic advance as a percent of the mean (GAM) was estimated and categorized by the following formula:

(2) $$Genetic\;advance\left(GAM\%\right)=\frac{K*H*p}{Mean}\;\times \;100$$

where K = 2.06 at 5% selection intensity; H = Heritability; P = Phenotypic standard deviation. The GAM was categorized into three classes: <10% (Low), 10–20% (Moderate), and > 20% (High). Pearson’s correlation coefficients between grain yield-related traits were calculated using the Corrplot R package v0.92 [24], and the combined BLUEs for two years of evaluation were used for QTL analysis.

### 2.4. Leaf Sampling, DNA Extraction, and Genotyping

Two weeks after seedling emergence, the newly expanded young middle leaflet of the trifoliate leaf was sampled per plant and placed in ziplock bags containing silica gel for desiccation according to Intertek Agritech laboratory’s protocol [27] and kept in the cold room of the cowpea breeding unit at IITA, Ibadan, Nigeria. Samples were sent to Intertek Sweden for DNA extraction and forwarded to the Diversity Arrays Technology (DArT) facility for genotyping. Genotyping was conducted using DArTag technology, a targeted genotyping method that has the capacity to genotype samples with specific or selected sets of SNP markers (https://www.diversityarrays.com/services/targeted-genotying/ (accessed on 5 February 2025)).

Genotyping was accomplished using the cowpea mid-density genotyping panel V1.0 [21]. This panel constitutes a subset of 2602 quality SNPs derived from the 51,128-SNP Cowpea iSelect Consortium Array [22]. The criteria used for marker selection included (1) iSelect missing data rate of less than 5%, (2) iSelect data MnAF greater than 0.2, and (3) uniform distribution along the genetic linkage groups [21]. The SNPs meeting these criteria were included in the DArTag test set for 318 (316 RILs and 2 parents) DNA samples. The DArTag genotyping was achieved utilizing special molecular probes designed to target small regions containing sequence variants. These targeted regions were subsequently amplified, and concurrently, sample-specific barcodes were attached. The resulting libraries were then subjected to sequencing on next-generation sequencing (NGS) platforms, Illumina Hiseq2500/Novaseq, with 1,200,000 reads per sample. The resulting sequences were processed using DArT PL’s proprietary pipeline, which includes aligning the sequences to fragments of the IITA cowpea variety IT97K-499-35 reference genome, which can be accessed on Phytozome (https://phytozome-next.jgi.doe.gov/info/Vunguiculata_v1_1 Vigna unguiculata v1.1, (accessed on 15 January 2025)) [2]. Guided by the DArTag oligos from the panel, allele calling was performed based on counts of alternative alleles for each sample and marker.

### 2.5. Linkage Map Construction

A linkage map construction based on SNP markers was carried out using the QTL IciMapping v.4.2 software MAP function [28]. Before carrying out the construction, SNP data conversion was performed to remove those showing no polymorphism between parents or among progenies or missing in the two parents. The function binning (BIN) was used to delete redundant markers and those with high missing rates. The segregation pattern of all the markers was evaluated based on chi-square (X2) values by assessing significant deviations from the expected Mendelian ratio of 1:1 for F6:7 RIL populations. Markers that showed statistically significant segregation distortions at a 5% probability level were excluded from further analysis. The genetic distances between markers and their order in the linkage group were estimated using Kosambi’s mapping function [29].

### 2.6. QTL Analysis and Candidate Genes Identification

QTLs were identified using the inclusive composite interval mapping (ICIM-ADD) function for all the evaluated traits. During the QTL mapping process, some critical parameters were set. These included 1000 shuffles for estimating the critical values of LOD at *p* = 0.05 of the probability value (*p*-value) for the permutation option to declare the presence of a significant main-effect QTL. The QTLs explaining 10% or more of the phenotypic variation (PVE ≥ 10%) were considered major, while those explaining less than 10% were classified as minor. To identify candidate genes associated with the measured traits, the flanking markers of the QTLs were selected and compared to the cowpea reference genome (www.phytozome.net), and the interPro portal was used for gene models along with their functional annotations.

## 3. Results

### 3.1. Phenotypic Variation

**Figure 1:**
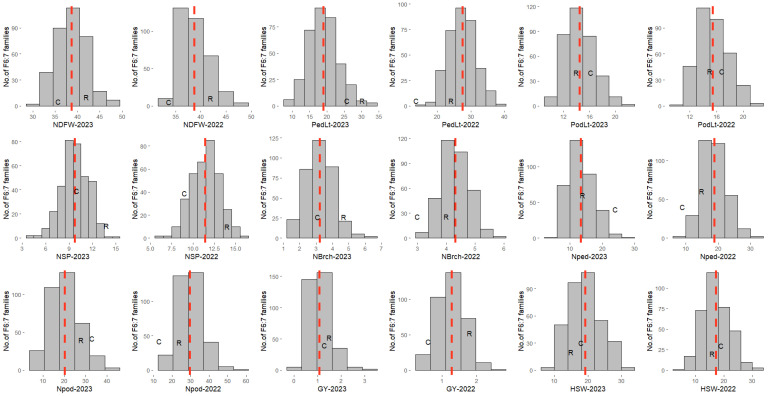
Frequency distributions of the number of days to flower, yield, and related traits in F_6:7_ RIL population for two years (2022 and 2023). Red lines show the population mean, while letters R and C represent the mean values of the parents, viz., RP270 and CB27, respectively. NDFW = days to first flowering, PedLt = peduncle length, PodLt = pod length, NSP = number of seeds/pod, NBrch = number of branches/plant, Nped = number of peduncles/plant, Npod = number of pods/plant, HSW = hundred-seed weight, GY = grain yield/plant.

The RILs and their parental lines were evaluated for nine traits whose frequency distributions showed continuous variation, nearly fitting the expected normal distribution across the two years (Figure 1).

Means, ranges (minimum and maximum value), standard errors, skewness, kurtosis, broad-sense heritability (H^2^), genotypic, phenotypic, and environmental variances, as well as coefficient of variations (CV%) associated with the traits are presented in (Table 1). The parental line RP270 exhibited higher values than CB27 for the number of days to first flowering, peduncle length, number of seeds per pod, number of branches per plant, grain yield per plant, and number of peduncles per plant in both years. However, line CB27 displayed slightly higher values than RP270 for 100-seed weight and pod length in both years. The ranges of phenotypic values of several RILs extended in both directions beyond those of their parents for all the studied traits, indicating transgressive segregations in the population (Figure 1). The RIL population showed different degrees of distribution for all the traits, with skewness and kurtosis mostly <1, which is typical of quantitative traits, thus indicating that this population is suitable for QTL mapping. Heritability in a broad sense (H^2^) in each year was above 60% for all the studied traits, except the number of branches per plant, suggesting high heritability for the traits. This observation also indicates that the phenotypic variation among the RILs was mainly genetic. However, the PCV values were higher than GCV for all nine traits, indicating that they all interacted with the environment to some extent. The grain yield (GY) was observed to show the highest phenotypic coefficient of variation and the number of days to first flower the lowest. The phenotypic coefficient of variation was found to be greater than their corresponding environmental coefficients of variation for all traits (Table 1).

The analysis of variance (ANOVA) for combined data of both years presented in Table 2 shows significant (*p* < 0.001) genotype mean squares for all traits, and the environmental mean squares were significant (*p* < 0.001) for all the traits except the number of days to first flower and grain yield. One hundred-seed weight showed the highest heritability value, followed by pod length, while grain yield exhibited the lowest heritability.

### 3.2. Correlations Among Traits

**Figure 2:**
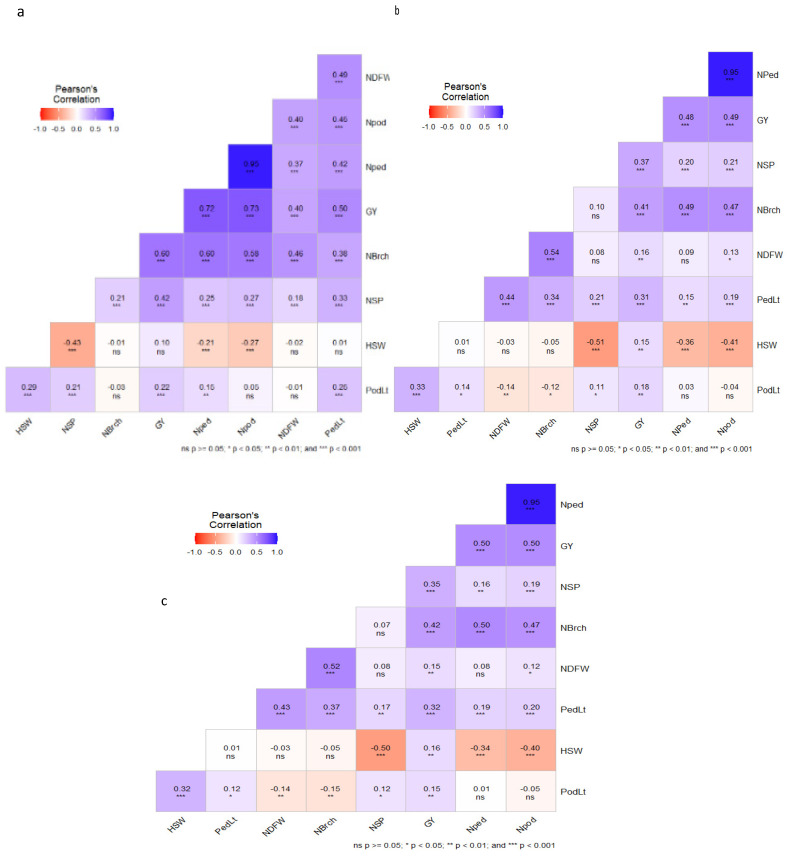
Correlation coefficients among yield and yield component traits. (**a**) year 2022, (**b**) year 2023, (**c**) combined data. Number of days to first flowering (NDFW), peduncle length (PedLt), pod length (PodLt), number of seeds/pod (NSP), number of branches/plant (NBrch), number of peduncles/plant (Nped), number of pods/plant (Npod), hundred-seed weight (HSW), and grain yield/plant (GY).

Pearson correlation analysis was carried out to identify trait relationships within and between traits. Hundred-seed weight displayed a significant and negative correlation with the number of seeds per pod in both years, with correlation coefficients of r = −0.43 and r = −0.51 for 2022 and 2023, respectively (Figure 2a,b). Meanwhile, days to first flowering exhibited significant positive correlations in both years with peduncle length and number of branches, with r2 values ranging from 0.34 to 0.54. Mean grain yield in both years was correlated with the number of seeds per pod, hundred-seed weight, number of peduncles per plant, number of pods per plant, and number of branches per plant, with r2 values ranging from 0.17 to 0.50 (Figure 2c).

### 3.3. Linkage Mapping

Following filtering of genotypic data, a total of 1083 high-quality SNP markers with confirmed positions were used for constructing a genetic linkage map that covered a total of 794.7 cM of cowpea genome (Table S1), with a cumulative average distance of 0.74 cM between adjacent markers. The number of SNP markers mapped on each of the 11 V. unguiculata linkage groups ranged from 65 for VuLG6 to 150 for VuLG3, spanning from 53.0 cM for VuLG11 to 120.1 cM for VuLG3, with a cumulative average of 72.25 cM. A variation in marker density was observed among the linkage groups. The highest density of 3.47 cM/Mb was found on VuLG4 and VuLG6, followed by VuLG11 (3.40 cM/Mb), while VuLG1 displayed the lowest marker density of 2.66 cM/Mb (Table S1).

### 3.4. QTL Mapping for Grain Yield and Related Traits

Detailed information on QTLs, including their peak positions, LOD scores, flanking markers, percent phenotypic variance explained, and estimated gene effects, are presented in Table 3.

**Figure 3:**
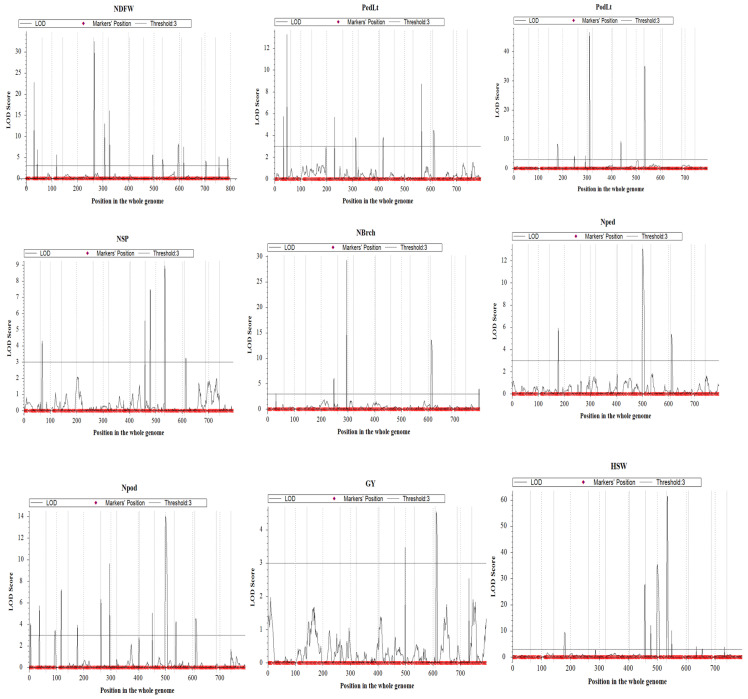
Manhattan plots of QTL mapping results. Each plot shows the LOD scores of markers for the respective traits. Significant QTLs are indicated by peaks that exceed the LOD score threshold, marked by a black dashed line. For the symbols indicating the names of respective traits, refer to Table 3 for explanation.

Thirty-one putative QTLs were identified under the additive model (ICIM-ADD) for nine yield and related traits distributed across all eleven chromosomes based on the critical LOD values set by permutation (Figure S1; Table 3). The Manhattan plots (Figure 3) provide visual representations of these findings, with LOD scores plotted against genomic positions. The highest number of QTLs were identified for the number of pods/plant (six), followed by the number of days to flower (five), pod length and hundred-seed weight (four each), peduncle length, number of branches per plant and number of peduncles/plant (three each), number of seeds/pod (two), and grain yield (one). A total of 7 of the 31 QTLs were found to be of major effects with PVE≥10%. There were two QTLs with major effects for hundred-seed weight, one each for the number of days to flower, pod length, number of branches per plant, number of peduncles per plant, and number of pods/plant (Table 3). The highest phenotypic variation explained by an individual QTL was 26.3% by *qHSW-7-1* for the hundred-seed weight identified on Chr7, and the lowest PVE was 1.8% by qPedLt-3-1 for peduncle length detected on Chr3.

Five QTLs, distributed across five chromosomes, were identified for the number of days to flower. However, the major and most significant QTL (*qNDFW-1-1*) for the number of days to flower on Chr1, with the highest LOD score of 22.8, PVE of 12.8% of total variation, and inherited from the later flowering parent RP270, showed an additive genetic effect (0.9), indicating the contribution of an allele that influenced this trait. One significant but minor QTL (*qNDFW-4-1*) detected on Chr4, with a LOD score of 13.0 and PVE of 7.2%, showed a negative additive effect (−0.7), indicating the contribution of a favorable earliness allele originating from CB27 that reduced the number of days to flower.

Three minor QTLs (*qPedLt-3-1*, *qPedLt-4-1*, and *qPedLt-9-1*) with LOD scores ranging from 3.0 to 4.5 and PVEs between 1.8% and 2.8% associated with peduncle length were detected on Chr3, Chr4, and Chr9. The QTLs found on chromosomes 3 and 9 showed positive additive effects of 0.5 and 0.6, respectively, indicating the contribution of favorable alleles from RP270 to increasing peduncle length, while that on chromosome 4 exhibited a negative additive effect (−0.6), indicating the allele contributing the decrease in peduncle length came from CB27.

A total of four QTLs were mapped for pod length: one major QTL (*qPodLt-8-1*), identified on Chr8, exhibited a LOD score of 33.5 and a PVE of 25.9%. It showed a positive additive effect (0.9), indicating the contribution of alleles from CB27. Three minor QTLs (*qPodLt-3-1*, *qPodLt-4-1*, and *qPodLt-6-1*) were located on Chr3, Chr4, and Chr6, respectively, exhibiting LOD scores between 3.5 and 8.1, with PVEs ranging from 2.3% to 5.2%.

There were two minor QTLs, *qNSP-6-1*, with a LOD score of 5.6 and PVE of 7.5%, and *qNSP-9-1* Chr9, with a LOD score of 3.1 and PVE of 4.3% of total variation, with effects on number of seeds per pod detected on Chr6 and Chr9, respectively. They showed a negative additive effect (−0.3), suggesting the contribution of the allele from CB27 in decreasing the number of seeds per pod. On the other hand, the total variation, with a positive additive effect (0.2), suggests the contribution of the favorable allele for a higher number of seeds per pod from RP270.

Three QTLs were mapped on three different chromosomes for the number of branches per plant. The major QTL (*qNBrch-9-1*) was found on Chr9 and exhibited a LOD score of 13.5 with a PVE of 10.5% of the total variation.

A total of three QTLs were identified on Chr3, Chr7, and Chr9 for the number of peduncles per plant. The major one, *qNped-7-1*, mapped on Chr7, had a LOD score of 13.0 and PVE of 15.0% of the total variation and a positive additive effect (1.3).

Six QTLs associated with the number of pods per plant were found on chromosomes Chr1, Chr2, Chr3, Chr4, Chr7, and Chr9. The QTL on Chr7 (*qNpod-7-1*) showed the highest LOD score of 13.9 and PVE of 10.5% of total variation, while that on Chr3 (*qNpod-3-1*) had the lowest LOD of 3.5, PVE of 2.4, and negative additive effect of -0.9.

Four QTLs associated with the hundred-seed weight were identified on Chr3, Chr7, Chr8, and Chr10. The two most significant QTLs were *qHSW-7-1* and *qHSW-8-1*, detected on Chr7 and Chr8, with LOD scores of 35.6 and 27.7, explaining PVE of 26.3% and 19.3% of total variation, respectively. These QTLs showed negative and positive additive effects, respectively, suggesting the contributions of alleles to decreasing or increasing 100-seed weight.

Only one minor QTL was identified on Chr9 (*qGY-9-1*) for grain yield. It had a LOD score of 4.5 and a PVE of 6.7%.

**Figure 4:**
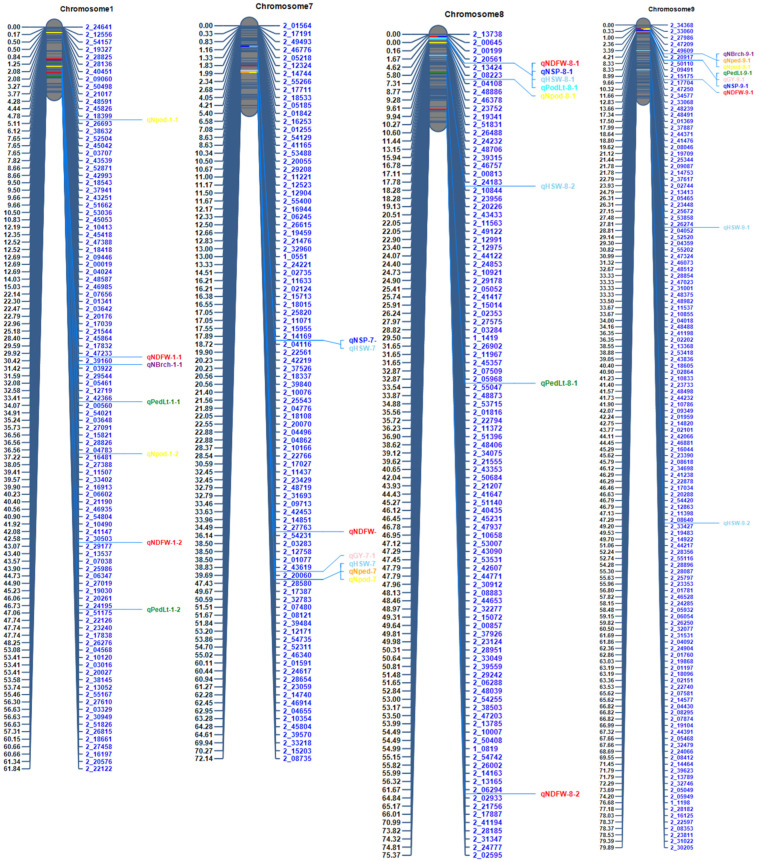
Cluster of QTLs associated with yield and related traits. The QTLs for traits are represented using their QTL names. For explanation of traits, see Table 1 footnote.

QTL clusters were identified across four chromosomes (Chr1, Chr7, Chr8, and Chr9), where multiple loci associated with yield and other traits are co-located, suggesting genes with pleiotropic effects (Figure 4).

### 3.5. Putative Candidate Genes for Yield and Related QTLs

The genes underlying the markers associated with QTLs for yield and related traits were retrieved and reported in Table S2. Based on gene ontology (GO) annotation, four key genes were retained based on their roles in controlling flowering time traits in grain crops. These genes include one on Chr1, AUX/IAA protein (*Vigun01g138700*); one on Chr4, Heat shock factor (HSF)-type (*Vigun04g159400*); one on Chr7, AUX/IAA protein (*Vigun07g131700*); and another on Chr8, B3 DNA binding domain (*Vigun08g019900*). Peduncle and pod lengths were analyzed as a measure of organ growth or elongation. Three genes were identified on Chr3 for peduncle length: one gene encoding the protein kinase domain (*Vigun03g015300*), one encoding glycosyl transferase family 14 (*Vigun03g015600*), and one encoding the NAC domain (*Vigun03g015600*). As pertains to pod length, four genes related to organ size increase, including pod length, were identified. One was identified on Chr3 (*Vigun03g233100*) encoding pentatricopeptide repeat, two on Chr4 encoding F-box domain (*Vigun04g057300*) and glycosyl transferase family 8 (*Vigun04g057700*), and one was identified on Chr8 (*Vigun08g206500*) coding for the protein kinase domain. One gene responsible for regulating plant architecture, including branch formation, by controlling the expression of genes participating in cell proliferation and differentiation was identified on Chr3 (*Vigun03g236400*), encoding transcription factor MYC. Four genes were reported for the number of peduncles per plant: two on Chr3, including transcription factor GRAS (*Vigun03g285400*) and lateral organ boundaries, LOB (Vigun03g285600), and two on Chr7, encoding small auxin-up RNA (*Vigun07g062500*) and transcription factor TCP (*Vigun07g074900*). Seven putative genes based on their roles in regulating the number of pods per plant were identified: one on Chr1, transcription factor GRAS (*Vigun01g035700*); one on Chr2, small auxin-up RNA (*Vigun02g123600*); two on Chr3, transcription factor GRAS (*Vigun03g285400*) and lateral organ boundaries LOB (*Vigun03g285600*); one on Chr4, transcription factor GRAS (*Vigun04g150300*); two on Chr7, cytokinin-activating LOG-family (*Vigun07g070900*), transcription factor, TCP (*Vigun07g074900*). Six key genes prioritized based on their roles in controlling seed size and fruit development in crop plants were identified. These include one gene on Chr3, serine-threonine/tyrosine-protein kinase catalytic domain (*Vigun03g271100*); two on Chr7, cytochrome P450 *(Vigun07g063300*), and F-box domain (*Vigun07g065300*); and three on Chr8, viz., WRKY domain (*Vigun08g206800*), ABC transporter-like (*Vigun08g208300*), and cytochrome P450 (*Vigun08g208600*).

## 4. Discussion

In the past thirty years, global cowpea production has expanded at an average annual rate of 5%, with a 3.5% yearly increase in cultivated area and 1.5% growth in yield, with the area expansion accounting for 70% of the total growth during this period [13,30]. To meet the projected global demand of approximately 11.2 million metric tons of grains by 2030, it is crucial to exploit the genetic diversity of this crop for a higher yield than what is obtainable from currently existing high-performing, farmer-preferred cultivars. Additionally, with the rapid growth in the global population, there is an urgent need to breed high-yielding cowpea varieties that adapt to climate change, which has become a major limiting factor for food production in many parts of the world. Frequency distributions for the traits measured showed no significant deviations from the corresponding normal distributions, indicating the quantitative nature of inheritance for the traits. There were appreciable variations in the ranges for all the measured traits, with values of several RILs extending in both directions beyond those of their parents, indicating transgressive segregations in the population. The RILs expressed more genetic variation and variation in gene expression than the two parents, thereby resulting in their having traits that are extreme in nature. Transgressive segregation suggests that some of the RILs contain a new combination of multiple genes with more positive or negative effects for the quantitative traits than were present in either parent. This segregation pattern could be a source of novel characters for some of the RILs. The presence of transgressive segregation has also been observed before in reports of [7,31].

Understanding the genetic basis of traits that impact yield is imperative for the development of improved varieties possessing the genes that enhance high productivity. According to Zaki et al. [7] cowpea yield can be improved by focusing on improving pod- and seed-related traits, which are direct contributors to the economic value of the crop. Among the yield and related traits considered in this study, number of peduncles per plant, number of pods per plant, number of seeds per pod, and 100-seed weight are the most relevant to consider in a cowpea improvement program aimed at increased grain yield.

Recent developments in plant genomics have led to new and enhanced breeding approaches, which have greatly expedited the breeding process [22,32,33]. The application of modern genomic tools and techniques has allowed plant breeders to develop superior varieties at a faster rate than conventional breeding methods, thereby increasing the frequency of releasing new, improved varieties to farmers. The new genomic tools facilitate the identification of specific regions or QTLs associated with desirable traits and the discovery of genes controlling both simple and complex trait expressions [9,13,34].

Detailed analysis of phenotypic data showed statistically significant variations among the RIL population in both years of evaluation. In this study, genotypic variances exceeded their corresponding environmental variances for all the measured traits. Similar observations have been reported, and the authors concluded that genotypic components contributed significantly to the total variations in such traits [35,36,37]. The estimated heritability of the evaluated traits was generally high (>60%) over the two years, aligning with previous studies [7,31]. In particular, the hundred-seed weight exhibited the highest heritability, thus agreeing with previous studies [13,38]. This indicates that progress in improving cowpea seed weight can be achieved consistently across different environments. The correlation analyses showed that improving cowpea grain yield is possible through selection for number of seeds per pod, number of peduncles per plant, number of pods per plant, and number of branches per plant. In this study, GCV and PCV values were quite close to each other for most of the traits, indicating low environmental influences.

QTL mapping is widely used to identify markers that are associated with desirable quantitative traits in crops. The efficiency and precision of QTL mapping largely depend on the diversity between parental lines used and the density of markers in the linkage map [39]. The quality of the genetic linkage map constructed plays a significant role in the accuracy of QTL identification.

Flowering time is a critical agronomic trait in cowpea, particularly in arid and semi-arid tropical regions that are drought-prone due to the reduction in the length of rainy seasons. Early flowering can serve as an effective drought escape mechanism, enabling genotypes to complete their reproductive cycle and mature before the onset of terminal drought [9,40]. Five QTLs were identified for the number of days to flowering, aligning with earlier studies [9,34,40,41,42]. The major effect QTL *qNDFW-1-1* for this trait might hold potential for deployment in cowpea breeding efforts using marker-assisted breeding strategies. Four key genes were retained based on their roles in controlling flowering time, such as the AUX/IAA protein on Chr1 and Chr7, which shows expression in various pathways, including the phytohormone signaling pathway, polyamine biosynthesis pathway, and flowering-related pathway [43]. The Heat shock factor (HSF)-type on Chr4 has been shown in Arabidopsis to repress flowering by interacting with FLOWERING LOCUS C (FLC) [44]. Furthermore, the B3 DNA binding domain on Chr8 promotes the floral transition in Arabidopsis during a long-day photoperiod through interactions with the flowering locus (FT) [45].

In cowpea, long peduncles are desirable as they enable pods to rise above the canopy, a characteristic that helps to reduce damage to the pods by the pod borer Maruca vitrata. Additionally, the raised pod position due to longer peduncles makes pod harvesting easier both manually and mechanically [10]. The three minor QTLs detected seem to be novel.

The major-effect QTL for pod length, *QTLqPodLt-8-1*, explained the highest phenotypic variance of 25.9%. This finding is consistent with earlier reports that identified QTL for pod length on Chr8 with a significantly highest phenotypic variance explained of 46.08% [9].

Increasing the number of seeds per pod in cowpea is a valuable trait that can significantly increase grain yield and economic value, aligning with food security and breeding programs’ objectives. Two minor QTLs were detected for this trait on Chr6 and Chr9. The QTL mapped on Chr9 was previously reported by [9,13,34].

Increasing the number of branches, peduncles, and pods per plant in cowpea is a promising strategy for enhancing potential grain yield. Three QTLs were mapped across three chromosomes (Chr3, Chr9, and Chr11) for the number of branches per plant. There is no record in the literature on QTL identification for the number of branches per plant in cowpea, making the present findings probably the first-ever report.

Three QTLs with effects on the number of peduncles per plant were identified. The one located on Chr9 was previously reported, while the QTLs mapped on Chr3 and Chr7 appear to be novel [13,30].

For the number of pods per plant, six QTLs were mapped on six chromosomes. However, Garcia–Oliveira et al. [13] reported QTLs with effects on the number of pods per plant on Chr8. Since none of the six QTLs detected in the present study was on Chr8, it can be concluded that they are novel, having not been reported previously. Genes identified as potentially influential in regulating organ growth, which can impact traits such as peduncle and pod length, number of pods, number of peduncles, and number of branches per plant, were prioritized. These genes include protein kinase domain, glycosyl transferase family 14, NAC domain, transcription factor TCP, small auxin-up RNA, pentatricopeptide repeat, and F-box domain, all of which encode transcriptional regulators involved in various aspects of plant growth and development, including gibberellin signal transduction, root radial patterning, axillary meristem formation, phytochrome, and resistance to disease in grain crops [46,47,48,49,50].

Large seeds play a major role in consumer preference, and a total of four QTLs were identified for hundred-seed weight. The main-effect QTL, qHSW-7-1, seems to be an interesting one, accounting for 26.3% PVE with a LOD of 35.6. It might be useful for breeding cowpea for high seed weight and seed size. In the past, QTLs for seed weight were also reported on the same chromosomes mentioned here [9,13,34,51,52,53]. Six putative genes have been identified for their roles in influencing seed size and fruit development in grain crops. Among them, serine-threonine/tyrosine-protein kinase (*Vigun03g271100*) is involved in regulating oil content in Arabidopsis seeds [54]. Cytochrome P450 (*Vigun07g063300*) plays an essential role in seed growth [55]. The F-box domain (*Vigun07g065300*) is linked to regulatory mechanisms for protein stability and is a key pathway for the degradation of most intracellular proteins [56]. Last, the WRKY domain (*Vigun08g206800*) is associated with the regulatory functions of WRKY transcription factors in seed development, germination, and seed vigor [57]. Only a single QTL with a minor additive effect was identified on Chr9 for grain yield.

It is worth mentioning that most of the evaluated traits were correlated with each other across the two years. The correlation responses between two or more variables can be explained at the molecular level by the presence of colocalized QTLs, which may contain specific genes with pleiotropic effects or groups of certain genes that coexist within the same genetic regions [58]. A significant aspect of this study is that QTL clusters affecting multiple traits were identified on Chr1, Chr7, Ch8, and Chr9, indicating potential pleiotropic effects as the genes controlling these traits are located in the same genomic regions. For instance, the co-localization reported on QTLs for multiple traits on Chr9 at position 5 cM, identified by the same markers including 2_20917 and 2_50110, with effects on peduncle length, number of branches (qNBrch-9-1), number of peduncles (qNped-9-1), number of pods (qNpod-9-1), and grain yield (qGY-9-1). The clustering of QTLs controlling multiple traits in cowpea is not common, as reported previously [13,59].

## 5. Conclusions

This study offers novel QTLs for many yield-related traits, and four notable QTL clusters were detected on different chromosomes. The cluster might have great potential for marker-assisted selection programs. Additionally, the findings of this study provide a basis for further gene mapping regulating cowpea grain yield-related traits. Evaluation of this RIL population across various agro-geographical regions would help validate the detected QTLs and identify stable QTLs for yield-related traits, which makes the application of marker-assisted selection possible in local cowpea breeding programs.

## Figures and Tables

**Table 1:** Mean values, ranges, variance components, and broad-sense heritability (H^2^) for days to first flowering, yield, and related traits.

		Parent (Mean)		RILS-RP270XCB27													
Trait	Season	RP270	CB27	Range	Mean	SE	Skew	Kurt	GV	PV	EV	GCV %	PCV %	ECV%	CV/%	H^2^%	GAM
NDFW	2022–23	42	33.7	29–50	38.7	0.11	0.2	−0.03	10.7	12.6	1.9	8.5	9.2	3.6	9.2	0.94	17.9
	2023–24	42	36	32–50	38.7	0.1	0.7	0.1	8.5	9.8	1.4	7.5	8.1	3	8.1	0.95	15.9
PedLt	2022–23	24.2	13.8	5.6–36.7	18.9	0.16	0.4	−0.1	15.4	27.4	12	20.7	27.6	18.3	27.7	0.81	46.1
	2023–24	30.2	25.8	8.7–45.4	27.5	0.16	0.2	0.1	10.3	28	17.7	11.7	19.3	15.3	19.3	0.65	25.7
PodLt	2022–23	15.2	16.3	7.8–23.9	14.5	0.08	0.5	0	4.2	6.4	2.1	14.2	17.4	10.1	17.4	0.88	31.5
	2023–24	14.5	16.6	9.30–23.4	15.4	0.08	0.4	−0.3	4.5	5.9	1.4	13.8	15.8	7.6	15.8	0.91	29.7
NSP	2022–23	14.8	9.4	3.2–17.7	9.7	0.07	0	−0.04	2.1	4.6	2.5	15	22.1	16.2	22.3	0.74	34
	2023–24	14.8	12.4	3.40-17.2	11.4	0.07	−0.3	−0.1	2.3	4.4	2.1	13.3	18.4	12.7	18.4	0.79	30.1
NBrch	2022–23	3.9	2.1	0.00–7	3.2	0.03	0.3	0.1	0.5	1.1	0.5	22.9	32.5	23	33.5	0.74	51.3
	2023–24	4.7	4.2	2.0.8.0	4.3	0.03	0.2	0.4	0.1	0.5	0.4	17.1	16.9	15.3	19.5	0.43	17.2
Nped	2022–23	14.7	8.9	3.5–30.5	13.4	0.15	0.7	0.5	11.6	22.4	10.8	25.4	35.3	24.5	35.5	0.78	57.4
	2023–24	18.7	21.5	5.20–44.8	18.8	0.17	0.5	0.6	9.2	26.9	17.7	16.1	27.5	22.3	28.7	0.64	37.7
Npod	2022–23	24.1	12.9	4.8–53	20.2	0.25	0.9	1	37.4	65	27.6	30.3	40	26.1	40.6	0.81	67.8
	2023–24	28.9	33.1	8.30–69.	29.6	0.27	0.5	0.8	28.6	70	41.4	18	28.2	21.7	29.8	0.68	41.8
HSW	2022–23	17.4	18.3	7.77–32.81	19.1	0.15	0.4	−0.3	22	24.7	2.7	24.5	26	8.5	26	0.96	51.7
	2023–24	15.5	17.2	4.52–35.43	17.2	0.15	0.4	0.1	20.4	22.9	2.5	26.3	27.8	9.2	27.8	0.97	55.4
GY	2022–23	1.8	0.6	0.10–3.76	1.1	0.02	1.3	2.7	0.2	0.3	0.1	40.1	51.7	32.6	52.2	0.83	88.9
	2023–24	1.5	1.4	0.10–3.1	1.3	0.02	0.3	−0.1	0.1	0.2	0.1	26.6	37.6	26.6	40.2	0.73	60.3

Days to first flowering (NDFW), peduncle length (PedLt), pod length (PodLt), number of seeds/pod (NSP), number of branches/plant (NBrch), number of peduncles/plant (Nped), number of pods/plant (Npod), hundred-seed weight (HSW), grain yield/plant (GY).

**Table 2:** Means, variance components, and broad-sense heritability H^2^ (%) estimates for measured traits (two years combined).

Trait	Mean	GV	EV	PV	GCV%	ECV%	PCV%	H^2^	MSG	MSE	MSGE
NDFW	38.7	8.08	1.65	9.73	7.35	3.33	8.07	0.89	54.413 ***	0.055ns	6.174 ***
PedLt	23.2	10.66	51.51	62.16	14.06	5.54	33.96	0.77	80.8 ***	6663.1 ***	19.2 ***
PodLt	15.0	4.05	2.25	6.30	13.45	8.59	16.77	0.91	25.947 ***	92.810 **	2.416 ***
NSP	10.6	1.88	3.75	5.63	12.95	12.15	22.42	0.80	13.469 ***	77.512 **	2.692 ***
NBrch	3.8	0.12	1.23	1.35	9.35	34.20	30.93	0.44	1.6370 ***	4.2329 *	0.9250 ***
Nped	16.1	3.53	30.66	34.18	11.66	7.99	36.28	0.40	51.141 ***	263.8 **	30.756 ***
Npod	24.9	14.28	84.66	98.93	15.18	5.17	39.97	0.50	166.91 ***	591.69 *	84.18 ***
HSW	18.2	20.78	4.56	25.34	25.07	7.07	27.68	0.97	123.931 ***	20.187 **	3.993 ***
GY	1.2	0.04	0.16	0.21	17.80	109.77	38.66	0.39	0.65486 ***	0.25218ns	0.40523 ***

Number of days to first flowering (NDFW), peduncle length (PedLt), pod length (PodLt), number of seeds/pod (NSP), number of branches/plant (NBrch), number of peduncles/plant (Nped), number of pods/plant (Npod), hundred-seed weight (HSW), grain yield/plant (GY), genotypic variance (GV), environmental variance (EV), phenotypic variance (PV), phenotypic coefficient of variance (PCV%), genotypic coefficient of variance (GCV%), heritability (H^2^), mean square genotype (MSG), mean square environment (MSE), mean square genotype x environment. *, ** and *** refers to statistical significance at the probability levels of 0.05, 0.01 and 0.001 respectively.

**Table 3:** List of putative QTLs for number of days to flower, grain yield, and related traits.

Trait	QTL	Chr	Position	L-Marker	R-Marker	LOD	PVE (%)	Add
Number of days to flower	*qNDFW-1-1*	1	30	2_47233	2_39160	22.8	12.8	0.9
	*qNDFW-4-1*	4	45	2_51619	2_53566	13.0	7.2	−0.7
	*qNDFW-5-1*	5	5	2_21129	2_12640	16.1	8.7	0.7
	*qNDFW-7-1*	7	35	2_27763	2_54231	5.6	3.0	0.4
	*qNDFW-8-1*	8	65	2_02933	2_21756	7.9	4.0	−0.5
Peduncle length	*qPedLt-3-1*	3	55	2_00783	2_13641	3.0	1.8	0.5
	*qPedLt-4-1*	4	50	2_25413	2_24694	3.7	2.3	−0.6
	*qPedLt-9-1*	9	5	2_20917	2_50110	4.5	2.8	0.6
Pod length	*qPodLt-3-1*	3	105	2_39184	2_07809	3.5	2.3	0.3
	*qPodLt-4-1*	4	30	2_40244	2_45153	4.3	2.8	−0.3
	*qPodLt-6-1*	6	35	2_47637	2_18342	8.1	5.2	−0.5
	*qPodLt-8-1*	8	5	2_13424	2_08223	33.5	25.9	0.9
Number seeds/pod	*qNSP-6-1*	6	55	2_00324	2_18825	5.6	7.5	−0.3
	*qNSP-9-1*	9	5	2_20917	2_50110	3.1	4.3	0.2
Number of branches/plant	*qNBrch-3-1*	3	105	2_39184	2_07809	5.6	4.1	−0.1
	*qNBrch-9-1*	9	5	2_20917	2_50110	13.5	10.5	0.2
	*qNBrch-11-1*	11	50	2_25038	2_17139	4.0	2.7	−0.1
Number of peduncles/plant	*qNped-3-1*	3	35	2_31831	1_0718	5.5	5.9	−0.8
	*qNped-7-1*	7	40	2_20060	2_28580	13.0	15.0	1.3
	*qNped-9-1*	9	5	2_20917	2_50110	5.3	5.9	0.8
Number of pods/plant	*qNpod-1-1*	1	5	2_18399	2_26693	4.1	2.8	−1.0
	*qNpod-2-1*	2	55	2_54989	2_21931	7.0	4.9	−1.3
	*qNpod-3-1*	3	35	2_31831	1_0718	3.5	2.4	−0.9
	*qNpod-4-1*	4	0	2_20652	2_03897	5.4	3.8	−1.1
	*qNpod-7-1*	7	40	2_20060	2_28580	13.9	10.5	2.0
	*qNpod-9-1*	9	5	2_20917	2_50110	4.5	3.3	1.1
Grain yield/plant	*qGY-9-1*	9	5	2_20917	2_50110	4.5	6.7	0.1
Hundred-seed weight	*qHSW-3-1*	3	40	2_16043	2_07722	9.0	5.5	−0.8
	*qHSW-7-1*	7	40	2_20060	2_28580	35.6	26.3	−1.8
	*qHSW-8-1*	8	5	2_13424	2_08223	27.7	19.3	1.5
	*qHSW-10-1*	10	45	2_33719	2_20455	3.8	2.3	0.5

*q* QTL designation followed by a symbol of the trait name and number of the chromosome location of the QTL. Chr: chromosome; Pos: position of the QTLs. L-marker: left marker; R-marker: right marker; PVE%: phenotypic variance (%) explained by the QTL; Add: additive effect.

## Data Availability

The original contributions presented in the study are included in the paper/Supplementary Materials. For any additional questions, please reach out to the corresponding author.

## Supplementary Materials

The following supporting information can be downloaded at https://www.mdpi.com/article/10.3390/genes16030247/s1, Figure S1: Linkage map displaying putative QTLs associated with yield and related traits. The QTLs for traits are represented using their QTL names (for the symbols indicating the positions of QTL for respective traits, see the trait column in Table 3 for explanation); Table S1: Summary of SNP-marker distribution on cowpea linkage groups; Table S2: List of genes underlying the major-effect QTL regions identified for yield-related traits.
