# Supplementary material for: Quantitative Trait Loci Mapping for Yield and Related Traits in Cowpea
Source: Genes (Basel). 2025 Feb 21;16(3):247. doi: 10.3390/genes16030247 (PMC11941961; doi:10.3390/genes16030247)
Supplement: Supplementary file 1 [file Sup_Figure S1.pdf › Sup_Figure S1.pdf]

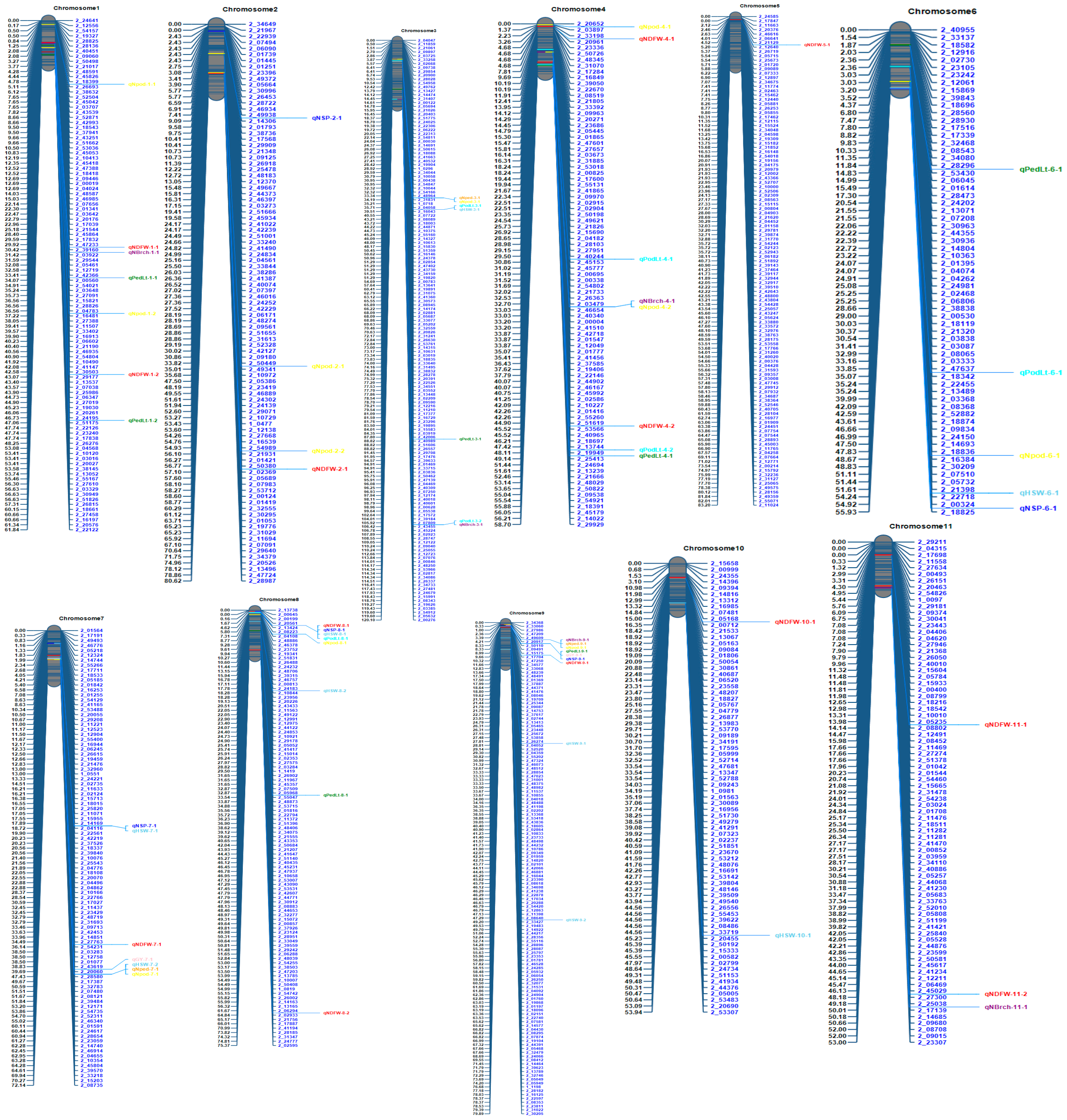

Figure S1: Linkage map displaying putative QTLs associated with yield, and related traits. The QTLs for traits are represented using their QTL names (For the symbols indicating the positions of QTL for respective traits, see trait column in Table 3 for explanation)
